# Diverse modulation of *spa* transcription by cell wall active antibiotics in *Staphylococcus aureus*

**DOI:** 10.1186/1756-0500-5-457

**Published:** 2012-08-25

**Authors:** Lene N Nielsen, Michael Roggenbuck, Jakob Haaber, Dan Ifrah, Hanne Ingmer

**Affiliations:** 1 Department of Veterinary Disease Biology, University of Copenhagen, Copenhagen, Denmark

**Keywords:** *Staphylococcus aureus*, Subinhibitory concentrations, Antibiotics, Virulence, Protein A (*spa*), Biofilm formation

## Abstract

**Background:**

The aim of this study was to investigate the effect of various classes of clinically relevant antibiotics at sub-lethal concentrations on virulence gene expression and biofilm formation in *Staphylococcus aureus.*

**Findings:**

*LacZ* promoter fusions of genes related to staphylococcal virulence were used to monitor the effects of antibiotics on gene expression in a disc diffusion assay. The selected genes were *hla* and *spa* encoding α-hemolysin and Protein A, respectively and RNAIII, the effector molecule of the *agr* quorum sensing system. The results were confirmed by quantitative real-time PCR. Additionally, we monitored the effect of subinhibitory concentrations of antibiotics on the ability of *S. aureus* to form biofilm in a microtiter plate assay. The results show that sub-lethal antibiotic concentrations diversely modulate expression of RNAIII, *hla* and *spa*. Consistently, expression of all three genes were repressed by aminoglycosides and induced by fluoroquinolones and penicillins. In contrast, the β-lactam sub-group cephalosporins enhanced expression of RNAIII and *hla* but diversely affected expression of *spa*. The compounds cefalotin, cefamandole, cefoxitin, ceftazidime and cefixine were found to up-regulate *spa*, while down-regulation was observed for cefuroxime, cefotaxime and cefepime. Interestingly, biofilm assays demonstrated that the *spa*-inducing cefalotin resulted in less biofilm formation compared to the *spa*-repressing cefotaxime.

**Conclusions:**

We find that independently of the cephalosporin generation, cephalosporins oppositely regulate *spa* expression and biofilm formation. Repression of *spa* expression correlates with the presence of a distinct methyloxime group while induction correlates with an acidic substituted oxime group. As cephalosporines target the cell wall penicillin binding proteins we speculate that subtle differences in this interaction fine-tunes *spa* expression independently of *agr*.

## Findings

### Background

Small molecules, such as antibiotics, are ubiquitous in the environment whether they originate directly from producing microorganisms or are the waste products of human activities [1,2]. While the antimicrobial activity of antibiotics target basic cellular functions like DNA, protein or cell wall synthesis they also affect other processes such as virulence gene expression [3,4]. β-lactam-containing penicillins and cephalosporins target transpeptidase and transglycosylase domains of the bacteria. They act as pseudosubstrates and acylate the active sites of the transpeptidases (also termed penicillin-binding proteins or PBPs) and have been widely used for treating infections including *S. aureus*[5]*.*

*S. aureus* is a serious human pathogen that causes many different types of illnesses ranging from enterotoxin mediated food intoxications to more severe infections such as endocarditis, pneumonia, osteomyelitis and toxic shock [6]. The pathogen is also one of the leading causes of biofilm-associated infections that typically are chronic and frequently occur in hospitals [7]. One of the key virulence factors often studied is α-hemolysin encoded by *hla.* α-hemolysin is a pore-forming toxin that targets red and white blood cells among other cell types [8,9]. While toxins and degradation enzymes are produced in stationary phase, the surface located virulence factors, including the IgG binding Protein A encoded by *spa*, are expressed in exponential phase. From mouse models of *S. aureus* infections, Protein A is known to be involved in development of pneumonia [10,11]. The protein has been proposed to act together with the Panton-Valentine leukocidin (PVL) to cause the severe inflammation and tissue damage seen in necrotizing pneumonia [12]. The *agr* quorum sensing system is the main regulator of virulence in *S. aureus* and controls the expression of at least 70 genes [13-15]. The effector molecule RNAIII inversely regulates *hla* (up) and *spa* (down) in response to increasing cell density [16].

The multitude of virulence factors contributing to the pathogenesis of *S. aureus* have spurred interest in how sub-lethal concentrations of antibiotics affect their expression and possibly modulate the outcome of infection. Exposure to macrolides, aminoglycosides and clindamycin reduces *hla* expression [17,18] whereas β-lactams and fluoquinolones increase transcription of *hla*[4]. The β-lactams have been used widely to treat *S. aureus* infections and here penicillins, cephalosporins and the carbapenem imipenem all increased *hla* expression with the monobactam aztreonam being the only exception [4]. For the cephalosporines this stimulation is likely to involve the SaeRS two-component system as the haemolytic activity induced by cefoxitin was abolished in the absence of *saeRS*[19]. However, a microarray gene expression analysis revealed that additional factors might be involved in the activation of *hla* expression by the cephalosporin cefoxitin [19]. *spa* transcription was examined in response to cell wall active antibiotics, including penicillins, cephalosoprins, carbanems and glycopeptides [20]. Induction levels varied between closely related antibiotics and the authors proposed that a chlorine substitution or cephalosporin generation could be responsible for the opposite effect of various penicillins on *spa* expression [20].

Although only addressed in a few cases, it appears that several unrelated types of antibiotics commonly either enhance or reduce expression of all the tested virulence genes no matter whether they encode cell surface or the secreted virulence factors. As these groups of virulence factors commonly are oppositely controlled by *agr* this observation indicates that the altered expression elicited by antibiotics is independent of *agr*. To address this issue more systematically we examined the expression of RNAIII*, hla* and *spa* for several classes of antibiotics using an agar-based reporter fusion assay recording the transcriptional activity of the corresponding promoter fusions. Also we have studied in details, the diverse modulation of *spa* transcription by various cephalosporins.

## Results and discussion

### Antibiotics modulate RNAIII, *spa* and *hla* differently

**Figure 1:**
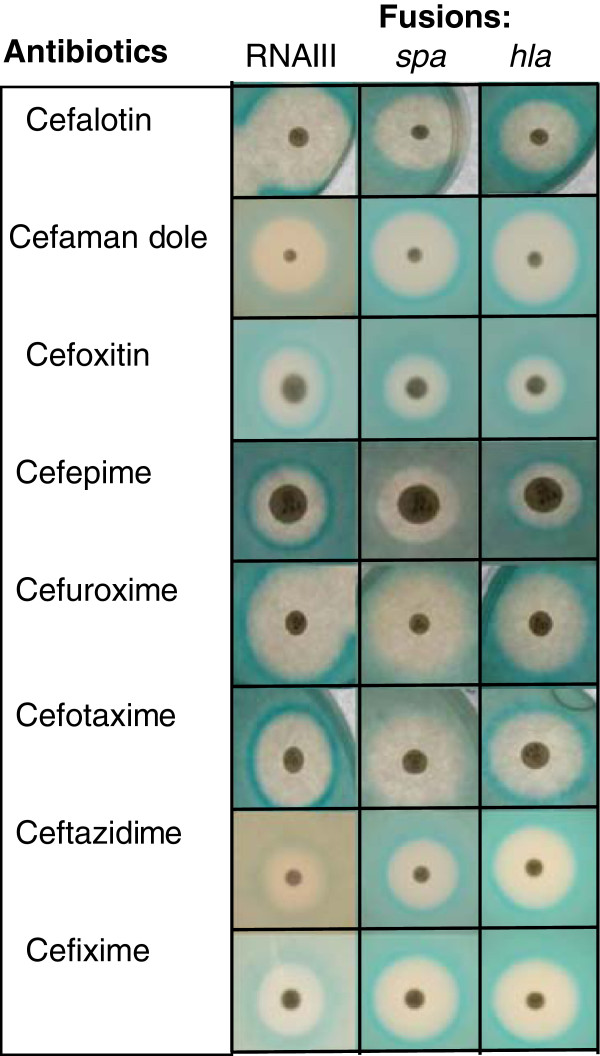
Representative results of disc diffusion assay monitoring transcriptional effects of cephalosporins.

We have used *S. aureus* strains carrying promoter l*acZ* fusions in *RNAIII, hla* and *spa* to study the transcriptional effects of antibiotics on virulence gene expression. We investigated the impact of a wide range of antibiotics including cell wall active antibiotics, that are the drug of choice when treating staphylococcal infections [20]. A disc diffusion assay was used for a broad screen of modulation of *RNAIII, spa* and *hla* transcription by various types of antibiotics (see Figure 1 for the cephalosporin representatives). A qualitative measure of gene expression was obtained by evaluation of the color intensity of the ring surrounding the inhibition zone (Figure 1). Results are listed in Table 1. The screen shows that the tested aminoglycosides reduced transcription of all reporter fusions, while fluorquinolones stimulated their transcription. On the other hand, while all β-lactams stimulated transcription of *RNAIII* and *hla* some enhanced and others reduced transcription of *spa*. The differentiated *spa* regulation was restricted to the cephalosporin group within the β-lactam family.

**Table 1:** Disc diffusion assay

**Class**	**Group**	**Antibiotics**	***rnaIII***	***spa***	***hla***
Aminoglycosides		amikacin	**-**	**-**	**-**
		kanamycin	**-**	**-**	**-**
		gentamycin	**-**	**-**	**-**
		spectinomycin	**-**	**-**	**-**
		streptomycin	**-**	**-**	**-**
		tobramycin	**-**	**-**	**-**
Fluoroquinolones		ciprofloxacin,	**+**	**+**	**+**
		enfrofloxacin	**+**	**+**	**+**
ß-lactams	Penicillins	ampicillin	**+**	**+**	**+**
		amoxicillin/clavulanic acid	**+**	**+**	**+**
		penicillin V	**+**	**+**	**+**
		oxacillin	**+**	**+**	**+**
		ticarcillin	**+**	**+**	**+**
	Cephalosporins	cefalotin (1^st^)	**+**	**+**	**+**
		cefamandole (2^nd^)	**+**	**+**	**+**
		cefoxitin (2^nd^)	**+**	**+**	**+**
		cefepime (2^nd^)	**+**	**-**	**+**
		cefuroxime (2^nd^)	**+**	**-**	**+**
		cefotaxime (3^rd^)	**+**	**-**	**+**
		ceftazidime (3^rd^)	**+**	**+**	**+**
		cefixime (3^rd^)	**+**	**+**	**+**

### Cephalosporin mediated changes in phenotype

**Figure 2:**
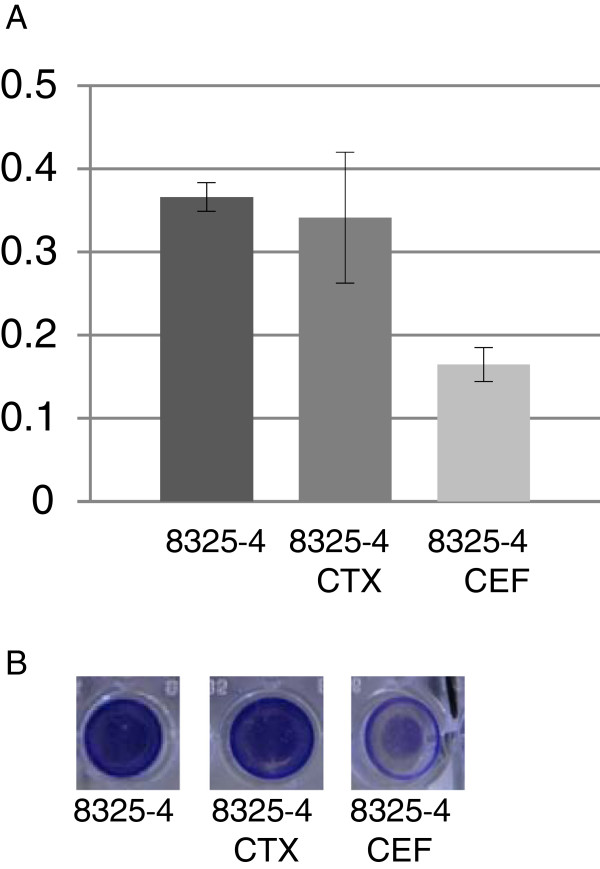
**Biofilm formation by *S. aureus *8325–4. A) Absorbance at 490 nm of 8325–4 and 8325–4 treated with cefotaxime (CTX) and cefalotin (CEF) respectively. B**) Crystal violet staining of biofilm of 8325–4 and 8325–4 treated with cefotaxime (CTX) and cefalotin (CEF) respectively.

Bacterial biofilm formation by *S. aureus* is a serious problem especially associated with the pathogenesis of implantable device-related infections, contributing to increased morbidity and mortality [21,22]. It is known that biofilm detachment is controlled by the *agr* quorum-sensing system [23-25] and because we observed opposite modulation of the *agr*-controlled *spa* gene when treated with the closely related antibiotics cefalotin and cefotaxime, we chose to investigate their influence on biofilm formation of *S. aureus* 8325*–*4. At sub-inhibitory concentrations both compounds reduced biofilm formation but the effect was most pronounced in the cefalotin exposed cultures (Figure 2). In our disc diffusion assay we observed increased expression of RNAIII when *S. aureus* was treated with cefalotin and cefotaxime (Table 1). Thus, the reduced biofilm formation correlates with an increased *agr* activity that previously has been shown to increase the detachment of *S. aureus* from biofilm and reduce biofilm [18,23,25]. The effect of sub-inhibitory cephalosporins on biofilm formation has been studied earlier with contradictory effect; here cefalotin and cephalexin respectively resulted in a denser biofilms [20,26]. These discrepancies may be explained by variations in methods used to study biofilm formation. Also, strain 8325–4 carries a mutation in *rsbU* that diminishes SigB activity [27]. Such strain variation is quite common in *S. aureus* and may explain differences between studies.

### Minor structural changes modulate *spa* differently

**Figure 3:**
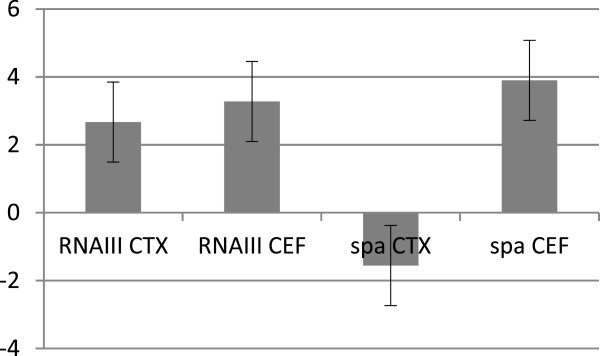
**Effect of cefalotin and cefotaxime on 8325–4 *spa *and *RNAIII *expression using qRT-PCR.** Cefalotin and cefotaxime were used in the concentrations 15.6 ng/ml and 8.3 ng/ml, respectively. Error bars represent standard deviation of biological replicates, n = 3.

**Figure 4:**
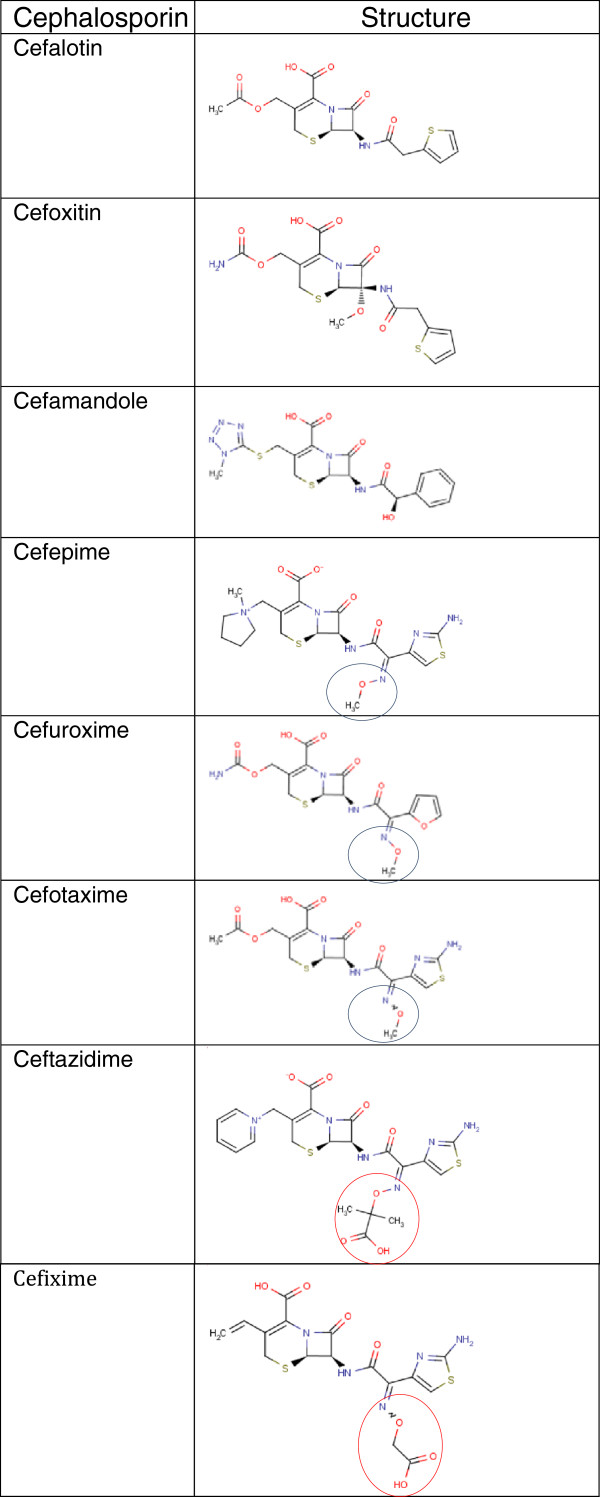
**Chemical structures of the Cephalosporins applied in this study.** The acidic functional group is circled in red, while the blue circled groups end in a space filling neutral group.

In our screen we had observed a differential modulation of *spa* expression when exposed to the closely related cephalosporins, cefalotin and cefotaxim. While both substances stimulated RNAIII transcription, cefalotin stimulated and cefotaxim repressed transcription of *spa*. This result was verified using qRT-PCR (Figure 3). Cefalotin and cefotaxime are 1^st^ and 2^nd^ generation cephalopsporins, respectively. Subrt *et al.* (2011) suggested that the generations of cephalosporines may cause the differences in *spa* expression [20]. In accordance with our data, they also found *spa* to be strongly stimulated by cefalotin (1^st^ generation) but reduced by cefoperazone (3^rd^ generation). To address the relationship between chemical structure, generation and *spa* expression in more detail we included additional cephalosporins with only minor structural differences (results summarized in Table 1). We tested cefoxitin and cefamandole, two 2^nd^ generation cefalosporins for their effect on *spa* regulation and found them to stimulate *spa* while cefepime, another 2^nd^ generation cephalosporin, down-regulated *spa* (Figure 1)*.* An additional 3^rd^ generation cephalosporin (ceftazidime) stimulated *spa*. Overall, these results indicate that the variable effects of cephalosporins on *spa* expression is not linked to the generation of the given cephalosporin but rather to specific structural features of the compounds. Examination of the chemical structures revealed that compounds reducing *spa* transcription, namely cefepime, cefuroxime and cefotaxime all have a distinct methyloxime group N-O-CH3, while the remaining have an acidic substituted oxime group, *i.e.* N-O-C(CH3)2CO2H or N-O-CH2-CO2H (Figure 4). These minor chemical changes could result in different binding affinities to a receptor molecule, such as the PBPSs. Cefalotin, cefamandole, cefoxitin and ceftazidime which stimulated *spa* have the greatest affinity for PBP1, PBP4 and PBP3 respectively [17,28-30] while cefotaxime and cefuroxime that down-regulated *spa* have highest affinity for PBP2 [29,31]. The observed down-regulation of *spa* by the latter two substances coincides with a stimulation of RNAIII (Table 1) correlating with *agr*-dependence, whereas the simultaneous stimulation of RNAIII and *spa* transcription by cefalotin, cefoxitin and ceftazidime is remarkable and must be considered *agr*-independent. In this study we have shown that the differences in *spa* transcription regulation are not due to cephalosporin generation but may be linked to small structural differences between the compounds. However, further studies are needed to elucidate the underlying mechanism.

## Conclusions

Our study shows that in *S. aureus*, sub-inhibitory concentrations of antibiotics diversely modulate virulence gene expression and that minor structural changes in the chemical structure have dramatic influence on their effect.

## Methods

### Strains and growth conditions

We used different *S. aureus* strains carrying *lacZ* promotor fusions in *RNAIII, hla* and *spa*. Before experiments, strains were incubated on tryptic soy agar (TSA) and grown overnight at 37°C. For the disc diffusion assay, an additional overnight incubation at 37°C in tryptic soy broth (TSB) aerated by shaking was performed. Strains are listed in Table 2.

**Table 2:** ***S. aureus *strains and constructs used in this study**

**Strain**	**Relevant characteristics**	**Reference**
8325-4	Wild-type strain cured of known prophages	[33]
JLA341	SH1000 *agr* (RNA III)::pAZ106 *agr*+	[34]
PC203	*spa*^*+*^*spa::lacZ Ey*^*R*^	[35]
PC322	*hla*^*+*^*hla::lacZ Ey*^*R*^	[35]

### Antibiotics, MIC- and sub-MIC determination

Antibiotics used in this study were: aminoglycosides (amikacin, kanamycin, gentamycin, spectinomycin, streptomycin, tobramycin), fluoroquinolones (ciprofloxacin, enfrofloxacin), and β-lactams (ampicillin, penicillin, oxacillin, amoxicillin/clavulanic acid, ticarcillin, cefalotin, cefepime, cefotaxime, cefuroxime, cefamandole, cefoxitin, ceftazidine, cefixime). Antibiotics were obtained from Sigma and Antimicrobial Susceptibility Testing discs (AST disc) from Oxoid Limited.

The minimal inhibitory concentrations (MIC) values were determined by broth microdilution assay as recommended by CLSI standards [32]. The sub-inhibitory concentrations were determined in Erlenmeyer flasks using OD_600_ to monitor growth of *S. aureus* 8325-4 [33]. A 2-fold serial dilution starting with the MIC value as the highest concentration was applied for every antibiotic. The highest concentration of a given antibiotic that did not visibly inhibit growth was chosen as the individual sub-inhibitory concentration. MIC values for cefalotin and cefotaxime were 10 μg/ml and 0.5 μg/ml respectively while the sub-inhibitory concentrations were 15.6 ng/ml and 8.3 ng/ml, respectively.

### Disc diffusion assay

Overnight cultures containing the transcriptional *lacZ* promoter fusions [34,35] were adjusted to OD_600_ = 0.0035 in 0.9% NaCl. One ml of the culture was placed in a petri-dish and mixed with 25 ml of 50°C warm TSA supplemented with Erythromycin (5 μg/ml) and X-gal (150 μg/ml). After solidification AST discs were placed on top of the plates and they were incubated for 16 hours at 37°C. Changes in gene expression were evaluated by visually judging the intensity of the blue color close to the inhibition zone compared to the background.

### Quantitative reverse transcriptase PCR

The effects of sub-inhibitory antibiotic exposure on gene expression was confirmed using quantitative reverse transcriptase PCR (qRT-PCR). Cefalotin and cefotaxime were used in the concentrations 15.6 ng/ml and 8.3 ng/ml, respectively. *S. aureus* 8325*–*4 was grown to OD_600_ = 1.0 and RNA purification was done using Qiagen RNeasy mini-prep according to the protocol. The RNA samples were further treated with DNase (Fermentas) before cDNA was made with a RT kit from Applied Biosystems. qRT-PCR was carried out in 96-well microtitre PCR plates (Sarstedt) using the primers listed in Table 3. The housekeeping pyruvate kinase (*pyk*) was used for normalization.

**Table 3:** Primers used in this study

**Primer name**	**Sequence**	**References**
* rnaIII-*F	GCACTGAGTCCAAGGAAACTAAC	This study
*rnaIII-* R	AAGCCATCCCAACTTAATAACC	This study
*spa*-F	CAAACGGCACTACTGCTGAC	This study
*spa*-R	CATGGTTTGCTGGTTGCTTC	This study
*pyk*-F	AGGTTGAACTCCCCAAACAA	This study
*pyk*-R	GCAGCCCAAGATTACAAAAA	This study

(+) up regulation, (−) down regulation of the given promoter::*lacZ* fusion.

### Biofilm assay

Relevant strains were grown to OD_600_ 0.2 at conditions and antibiotic concentrations as described above. Hundred μl pre-culture was added to a 96-well microtiter plate (Sarstedt) and incubated overnight at 37°C. Then, each well was washed three times with 200 μl physiological saline and stained with 0.1% crystal violet for 30 min. The crystal violet was washed out and each well washed three times with water. The amount of biofilm was determined by spectrophotometer at 490 nm after dissolving the crystal violet with 96% ethanol for 30 min. The test samples were made in triplicate and the experiment repeated three times.

## Competing interests

The authors declare that they have no competing interests.

## Authors’ contributions

LN was the primary author of the manuscript, a part of the disc assay screen, qPCR and biofilm assay. MR performed minimum inhibition concentration determination and the majority of disc assay screen. DI participated in analyzing the chemical structures. HI and JH designed the study. All authors contributed in writing and reviewing the manuscript. All authors read and approved the final manuscript.
